# Evolutionary Conserved Short Linear Motifs Provide Insights into the Cellular Response to Stress

**DOI:** 10.3390/antiox12010096

**Published:** 2022-12-30

**Authors:** Sergey P. Zavadskiy, Denis S. Gruzdov, Susanna S. Sologova, Alexander A. Terentiev, Nurbubu T. Moldogazieva

**Affiliations:** 1 Nelyubin Institute of Pharmacy, I.M. Sechenov First Moscow State Medical University (Sechenov University), 119991 Moscow, Russia; 2 Department of Biochemistry and Molecular Biology, N.I. Pirogov Russian National Research Medical University, 117997 Moscow, Russia

**Keywords:** alpha-fetoprotein, stress response, short linear motifs, bioinformatics, functional enrichment analysis

## Abstract

**Highlights:**

1. Hundreds of short linear motifs (SLiMs) that exhibit a high degree of sequence similarity to two biologically active sites of human alpha-fetoprotein (AFP) were identified.
2. The SLiMs of interest are ubiquitously distributed and found in proteins of both eukaryotic and prokaryotic species.
3. Proteins retrieved by sequence alignment belonged to various functional classes to be directly or indirectly involved in cellular response to stress.
4. Our findings provide insights into the common functions of evolutionary conserved SLiMs and putative involvement of AFP in response to external and internal stimuli during cellular adaptation during embryonic development and cancer.

**Abstract:**

Short linear motifs (SLiMs) are evolutionarily conserved functional modules of proteins composed of 3 to 10 residues and involved in multiple cellular functions. Here, we performed a search for SLiMs that exert sequence similarity to two segments of alpha-fetoprotein (AFP), a major mammalian embryonic and cancer-associated protein. Biological activities of the peptides, LDSYQCT (AFP_14–20_) and EMTPVNPGV (GIP-9), have been previously confirmed under in vitro and in vivo conditions. In our study, we retrieved a vast array of proteins that contain SLiMs of interest from both prokaryotic and eukaryotic species, including viruses, bacteria, archaea, invertebrates, and vertebrates. Comprehensive Gene Ontology enrichment analysis showed that proteins from multiple functional classes, including enzymes, transcription factors, as well as those involved in signaling, cell cycle, and quality control, and ribosomal proteins were implicated in cellular adaptation to environmental stress conditions. These include response to oxidative and metabolic stress, hypoxia, DNA and RNA damage, protein degradation, as well as antimicrobial, antiviral, and immune response. Thus, our data enabled insights into the common functions of SLiMs evolutionary conserved across all taxonomic categories. These SLiMs can serve as important players in cellular adaptation to stress, which is crucial for cell functioning.

## 1. Introduction

Short linear motifs (SLiMs) are evolutionarily conserved functional modules of proteins that represent amino acid stretches composed of 3 to 10 residues involved in recognition and targeting activities [1]. SLiMs function through transient interactions with a variety of binding partners, mostly, with globular protein domains of other proteins. Thereby, they are involved in protein–protein interactions, which underlie numerous cellular processes including signal transduction, metabolism, electron transfer, cell cycle, membrane transport, etc. [2]. Currently, it has become recognized that similar SLiMs can be found in numerous non-homologous, unrelated proteins recruited in common regulatory functions [3]. They exert evolutionary plasticity that has facilitated a rapid growth of their use and resulted in their ubiquitous distribution across a variety of organisms.

A growing body of data evidences that during the long evolutionary time, short amino acid segments undergo mutations and multiple events of re-use in a variety of non-homologous proteins [4]. Evolutionary events such as duplication, fusion, and recombination have been suggested to provide a mechanism for the reuse and successful incorporation of such stretches into multiple unrelated proteins [5,6]. Therefore, such reuse of pre-existing sequences is likely to offer an evolutionary advantage for functional proteins. 

Presumably, ancient proteins were quite short molecules and have evolved into contemporary large, globular, and functional proteins due to the incorporation of short peptide stretches [7]. Indeed, Eck and Dayhoff described the phenomenon of the incorporation of a prototype small iron–sulfur cluster-containing protein, ferredoxin, that is involved in electron transfer and redox regulation, into metabolic proteins [8]. This can happen at the very early stages of biochemical evolution due to the doubling of the prototype that was enriched in Ala, Asp, Pro, Ser, and Gly residues. The authors have identified two major functional types of primordial peptides composed of 9 to 38 residues in length. The first type represented nucleic acid-binding and ribosomal peptides, while another type was catalytic peptides that can coordinate metal ions, iron–sulfur clusters, nucleotides, and nucleotide-derived cofactors [9]. Therefore, identifying evolutionary conserved SLiMs with sequence similarity to the prototype peptides can be indicative of common ancestry and functional relationships [10,11]. 

Earlier, we identified a variety of human proteins that contain SLiMs with sequence similarity to two functionally important segments of human alpha-fetoprotein (AFP) [12]. These SLiMs have been proposed to orchestrate functioning of multiple non-homologous human proteins during embryonic development, redox regulation, and cancer progression. AFP is a major mammalian development- and cancer-associated protein that in human is composed of 609 amino acids organized in three structural domains (I, II, and III) [13]. Experimental data have evidenced that human and rodent AFPs are capable to bind metal ions and various hydrophobic ligands. 

Multiple linear segments with putative and experimentally confirmed functions have been identified to enable functional and structural mapping of human AFP [14]. The 34 amino acid-long stretch located in the domain III to encompass residues from 464 to 497 of full-length human AFP has been designated as growth-inhibitory peptide (GIP) and chemically synthesized, purified, and characterized [15,16]. The GIP has occurred to exert the inhibitory effects on mouse uterine cell proliferation and cancer growth in an MSF-7 cell line model [17]. Its *C*-terminal segments, EMTPVNPGV (GIP-9) that encompasses residues 489 to 497 has occurred to be one of the most biologically active segments of GIP [18]. Moreover, human AFP and derived peptides have been experimentally shown to reduce fetotoxicity of high doses of insulin and estrogens in murine and chick models [19]. 

Another AFP-derived peptide, LDSYQCT, is located in the domain I to encompass residues from 32 to 38 in the full-length protein. In the mature protein this segment encompasses residues from 14 to 20 and, consequently, it has been designated as AFP_14–20_ [20]. This heptapeptide has been shown to share a high degree of sequence similarity with a part of receptor-binding domain of epidermal growth factor (EGF). AFP_14–20_ has also been chemically synthesized and demonstrated the immunomodulatory effects in culture of human phytohemagglutinin (PHA)-activated lymphocytes [21]. Multiple analogs and fragments of this peptide have been obtained to display biological activity that correlated with amino acid composition that influences conformational changes in the protein backbone [22]. 

Here, we used the SLiM search approach based on local sequence alignment algorithms to retrieve proteins that contain short GIP-9-like and AFP_14–20_-like motifs from protein primary structure databases. We identified a vast array of proteins from all taxonomies, including bacteria, viruses, archaea, and eukaryotes that contain both SLiM types of interest. Amino acid composition analyses of all retrieved SLiMs allowed for the revealing of a high degree of sequence conservation and hotspot residues. Furthermore, we performed comprehensive Gene Ontology (GO) functional enrichment analysis and revealed that the both motif types can be identified in proteins involved directly or indirectly in cellular response to biotic and abiotic stress. Our data allow for the suggestion that these conserved motifs underlie the involvement of a vast array of proteins in cellular response to stress conditions. Also, AFP can be involved in cellular adaptation to oxidative, genotoxic, and metabolic stress during embryonic development and cancer growth.

## 2. Materials and Methods

### 2.1. Mapping of AFP_14–20_-like and GIP-9-like Peptides

Both biologically active peptides, AFP_14–20_ and GIP-9, were mapped on three-dimensional (3D) structure of human AFP in order to assess their structural features. For this purpose, we utilized the 3D structure of human AFP that we previously constructed by homology-based modelling with the use of Schrödinger software (release 2018-2) [23,24]. PyMOL, version 2.5, molecular graphics system was utilized for structure visualization (https://pymol.org/2/ (accessed on 21 January 2022)) [25].

### 2.2. Search for Short Linear Motifs

We carried out local sequence alignment with the use of both AFP-derived peptides, LDSYQCT and EMTPVNPGV, as queries for sequence similarity search. FastA suite [26] supported by the European Bioinformatics Institute of European Molecular Biology Laboratory (EMBL-EBI) (https://www.ebi.ac.uk/Tools/sss/fasta/ (accessed on 8 January 2022)) [27] was exploited. The alignment was performed against UniProtKB protein knowledgebase (https://www.uniprot.org/ (accessed on 8 January 2022)), both UniProtKB/Swiss-Prot (the manually annotated and reviewed) and UniProtKB/TrEMBL (the automatically annotated) sections [28]. No restriction in taxonomic categories was applied. GLSEARCH (version 36.3.8 h) algorithm provided the most optimal search for sequences that match the query peptides. Default parameters: BLOSUM50 matrix, gap open -10, gap extension -2, expectation value (E-value) upper unit 10 and lower unit 0 to obtain up to 500 alignments were utilized. 

### 2.3. Amino Acid Conservation Analysis

SLiMs obtained with the use of the FastA GLSEARCH algorithm were further subjected to amino acid substitution analysis. Amino acid substitutions at each position of all SLiMs were calculated as follows: N = *a*/*b* × 100%. Here, *a* is the quantity of a definite residue at a definite position and *b* is the total number of SLiMs. All SLiMs including those aligned to AFP itself from all species and uncharacterized and hypothetical proteins were taken into account. Graphical representation of the amino acid conservation was performed with the use of the WebLogo3 (http://weblogo.threeplusone.com/create.cgi (accessed on 5 February 2022)) tool [29].

### 2.4. Functional Classification of Retrieved Proteins

All proteins extracted from the both Swiss-Prot and TrEMBL sections of UniProtKB database were subjected to GO term-based functional classification [30] in both the molecular functions and biological processes categories (http://geneontology.org/ (accessed on 14 May 2022)). These included all retrieved proteins from both prokaryotic and eukaryotic taxonomies. Since TrEMBL is a large section that contains automatically annotated proteins, a cut-off of E-value 0.1 and identity degree of 57.1% for AFP_14–20_-like motifs and E-value 0.1 and identity degree of 55.6% for GIP-9-like motifs were applied for alignments against this section of UniProtKB. In addition to UniProtKB, InterPro (https://www.ebi.ac.uk/interpro/ (accessed on 7 June 2022)) protein family resource was used for functional annotations of the retrieved proteins [31].

### 2.5. Gene Set Enrichment Analysis

For further gene set enrichment analysis, two lists of genes coding for the retrieved proteins containing either AFP_14–20_-like or GIP-9-like motifs were manually created. The UniProtKB-IDs were used and when needed they were converted into Ensembl gene IDs and STRING-db proteins IDs. These datasets were used as backgrounds for GO enrichment analysis. The created lists were first uploaded into PANTHER classification system (http://pantherdb.org/ (accessed on 17 May 2022)) of the Gene Ontology resource [32]. The R/Bioconducter packages in graphical ShinyGO v0.75 suite (http://bioinformatics.sdstate.edu/go/ (accessed on 24 May 2022)) was utilized [33] for further functional enrichment analysis. Characteristics of a list of genes were compared with other genes of the whole genome (background) and Student’s t-test was applied. Additionally, the gProfiler functional enrichment analysis [34] resource (https://biit.cs.ut.ee/gprofiler/ (accessed on 28 May 2022)) was utilized. Here, the gSCS statistical threshold to be equal to 0.2 and ENTREZGENE_ACC numerical IDs to extract all known gene sets were exploited.

## 3. Results

### 3.1. Biologically Active Peptides Are Located on AFP Surface

**Figure 1:**
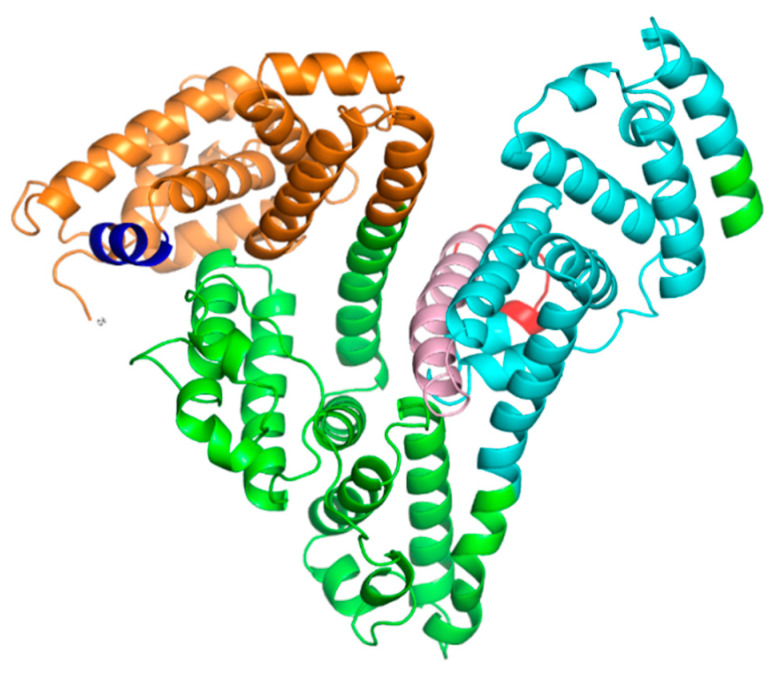
The overall architecture of AFP is represented by a U-shaped structure composed of three domains: I (orange, residues 19–210), II (green, residues 211–402), and III (cyan, residues 403–601). Two functionally important segments, AFP_14–20_ with sequence LDSYQCT (residues 32–38, colored in blue) and GIP-9 with sequence EMTPVNPGV (residues 489–497, colored in red) that is a part of GIP-34 (residues 464–497, colored in pink), respectively, are shown.

Figure 1 depicts the overall U-shaped architecture and 3D organization of human AFP with secondary structure elements represented by alpha-helices and loops with no beta-strands. Visualization of the obtained structure showed that the two distinct functionally important segments of human AFP with experimentally confirmed biological activities, AFP_14–20_ and GIP-9, are located on the protein surface to be accessible to the solvent and/or protein binding. 

The fist peptide segment is located in the domain I, close to *N*-terminus, and arranged in α-helical conformation. The second segment encompasses *C*-terminal part of GIP-34 peptide that occupies the most prolonged α-helical stretch in the domain III. Only a little part of secondary structure elements of the GIP-9 peptide is arranged in α-helix, while the remaining part represents a disordered region, and this can have a role in its functionality. 

### 3.2. Proteins Containing SLiMs of Interest Are Biologically Diverse

Local sequence alignment enabled retrieval of 464 proteins from Swiss-Prot section of UniProtKB database and 500 proteins from its TrEMBL section (with maximum E-value 6.9 × 10^-4^) that contain SLiMs with sequence similarity to LDSYQCT peptide. They covered proteins from a wide range of taxonomic categories and included uncharacterized and hypothetical proteins. Table 1 shows the most representative proteins from various species aligned with LDSYQCT sequence and the alignment E-values: the lower the E-value, the higher the statistical significance of the alignment. In the alignment column, the upper sequence is a query, whereas the lower sequence is from the retrieved protein. Proteins that contain AFP_14–20_-like motifs play various biological roles including transcriptional and translational regulation, oxidoreductase and electron transfer activity, protein quality control, host–pathogen interaction, biotic and abiotic stress response, and component of ribosomes and the toxin–antitoxin system, etc.

SLiMs with sequence similarity to EMTPVNPG octapeptide were identified in 258 proteins from the Swiss-Prot section and 500 proteins from the TrEMBL section (with maximum E-value 4.3 × 10^−2^) of UniProtKB database. These proteins covered all taxonomic categories and included AFP from different biological species, uncharacterized and hypothetical proteins. Table 2 contains the most representative proteins aligned to GIP-9 segment and the alignment E-values. Proteins that contain GIP-9-like motifs also have a wide range of biological roles including involvement in cell signaling, transcriptional regulation, metabolic processes, response to chemicals, immune response, electron transfer, etc.

### 3.3. SLiMs of Interest Are Enriched in Conserved Residues

**Figure 2:**
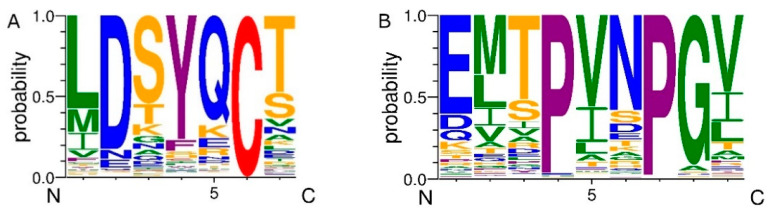
WebLogo representation of amino acid abundances at each position of (**A**) AFP_14–20_-like and (**B**) GIP-9-like motifs identified in proteins retrieved from UniProtKB database. The overall height of every stack indicates residue conservation at each position, while a symbol height within the stack indicates relative frequency of each residue at that position. Colors of symbols are as follows: hydrophobic and glycine—green, hydrophilic and positively charged—orange, negatively charged and their amides—blue, aromatic plus proline—purple, and cysteine—red.

After the exclusion of the same proteins from different taxonomies, 199 AFP_14–20_-like and 280 GIP-9-like unique motifs were identified. Furthermore, we assessed amino acid frequencies at each position of the unique SLiMs. The most conserved residue in AFP_14–20_-like motifs was cysteine (C) that comprises 100% of the total residue number at position 6. The second-most conserved residue was aspartic acid (D) that constituted 82.9% of all residues at position 2 and can be replaced, predominantly, by physicochemically similar asparagine (N) and glutamate (E). Two aromatic amino acids, tyrosine (Y) and phenylalanine (F), comprised 83.9% of all residues at position 4. While 57.3% of residues at position 1 were represented by leucine (L) that can be substituted for other hydrophobic residues—methionine (M), isoleucine (I), and valine (V). Serine (S) constituted 54.8% of all residues at position 3 to be replaced, mostly, by hydrophilic amino acids—T, K, and E. Hydroxyl group-containing residues, T and S, constituted 65.3% of all residues at position 7. Glutamine (Q) comprised 67.8% of all residues at position 5 to be replaced by charged and hydrophilic amino acids—lysine (K), aspartate (D), and arginine (R). These calculations with the application of a threshold of 5% resulted in the following notation for the consensus sequence: L[MIV]D[NE]S[TKE]Y[F]Q[KDR]CT[S]. Figure 2A graphically depicts the frequency of each amino acid at every position of the retrieved AFP_14–20_-like motifs.

As for GIP-9-like motifs, three most conserved positions were identified—4, 7, and 8. Positions 4 and 7 were occupied by proline (P) residue that comprised 96% and 98% of all residues, respectively. The third-most conserved residue was glycine (G) that comprised 92% of all residues at position 8. The least conserved position was 2, where methionine (35.4%) was the most frequent residue and could be replaced by other hydrophobic amino acids—L, I, and V. At position 1, glutamic acid residue constituted 60.4% of all residues to be replaced, more frequently, by D, Q, and K, which have similar physicochemical properties. Threonine (T) constituted 51.8% of all residues at position 3 to be replaced most frequently, by serine (S), a physicochemically similar residue (12.9%). Position 5 was occupied, mostly, by large hydrophobic residues—V (55.0%), I (20.4%), and L (7.9%). At position 6, asparagine (N) comprised 56.8% of all residues and the most significant replacement was for D and S, while position 9 was occupied by large hydrophobic amino acids—V (47.1%), L (12.1%), and I (18.2%). On the basis of the calculations, the following notation for consensus sequence was identified: E[DQ]M[LIV]T[S]PV[LI]N[DS]PGV[LI]. Figure 2B graphically depicts frequency of each amino acid at every position of the identified GIP-9-like motifs.

Therefore, both SLiM types of interest contain a large proportion of conserved amino acid residues indicating that they are evolutionarily preserved through all biological species starting from bacteria and viruses to higher eukaryotes. Interestingly, the consensus sequences were enriched in D, S, and P residues found in prototype peptides, which have been proposed to give rise to modern proteins.

### 3.4. Retrieved Genes Ubiquitously Exist

**Figure 3:**
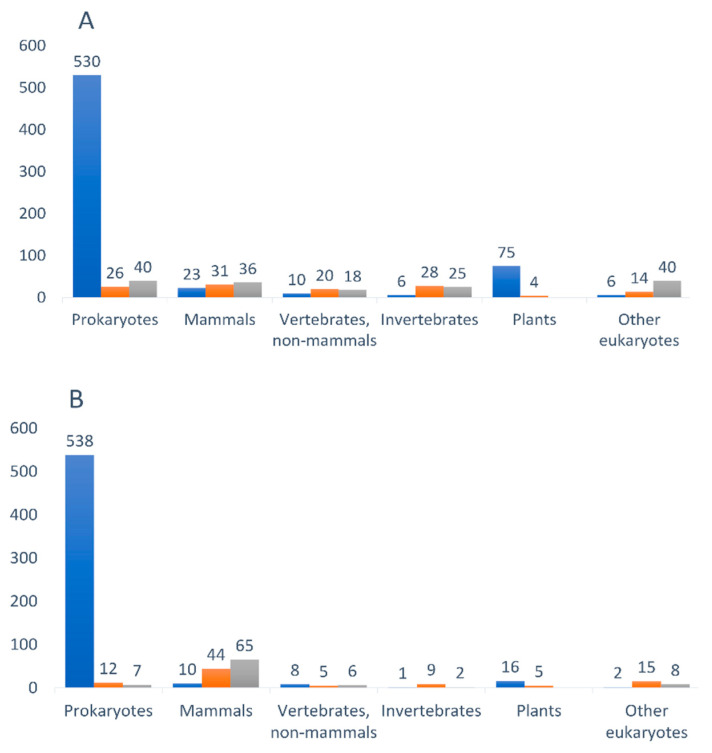
Diagram representations of taxonomic distribution of genes encoding proteins, which were retrieved from an UniProtKB database as aligned with (**A**) AFP_14–20_ and (**B**) GIP-9 segment of human AFP. Amounts of unique genes in each taxonomic category are shown above each column. Prokaryotes—bacteria (blue), viruses (brown), archaea (grey); mammals—*Homo sapiens* (blue), primates (brown), other mammals (grey); vertebrates—birds (blue), fishes (brown), amphibia (grey); invertebrates—reptiles (blue), insects (brown), nematodes (grey); plants—higher plants (blue), algae (brown); other eukaryotes—*S. cerevisiae* (blue), fungi (brown), mollusks, scorpions, spiders, etc., (grey).

Furthermore, we classified unique genes that code for proteins containing both SLiM types of interest on the basis of their belonging to any taxonomic category. We found that the retrieved genes are widely distributed among all taxonomic groups, including bacteria, viruses, archaea, and various invertebrate and vertebrate species, including mammals and primates. Figure 3A,B depicts the taxonomic distribution of unique gene coding for proteins with AFP_14–20_-like and GIP-9-like motifs, respectively.

Up to 64% and 74% of AFP_14–20_-like and GIP-9-like motifs, respectively, were found in bacterial proteins, while about 10% and 16% motifs, respectively, were identified in mammalian proteins. Some retrieved genes had orthologs in multiple biological species, therefore each of such genes was treated as a unique gene. For example, both SLiMs of interest were found in cytochrome c biogenesis protein CcmE and malate dehydrogenase from a wide range of bacterial species, while transmembrane protein TMEM258 was from various eukaryotic species (see Table 1 and Table 2).

### 3.5. Retrieved Proteins Are Functionally Diverse

**Figure 4:**
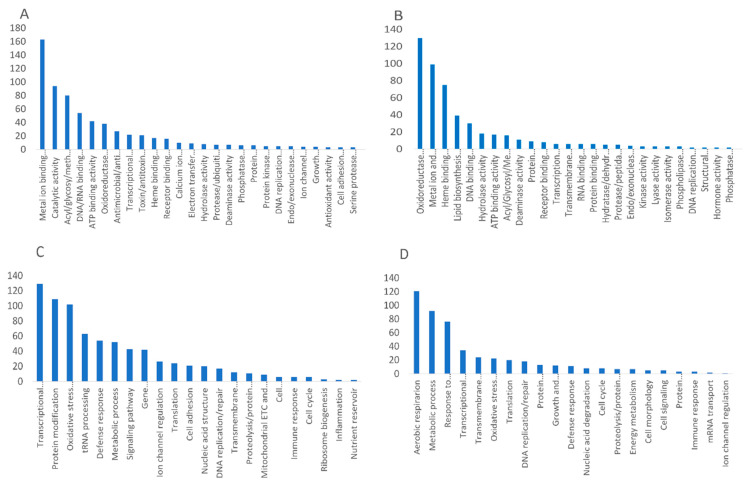
Categorization of proteins retrieved from an UniProtKB knowledgebase were performed in Gene Ontology. (**A**,**B**) Molecular function and (**C**,**D**) biological process terms and aligned with (**A**,**C**) AFP_14–20_ and (**B**,**D**) GIP-9 segments of human AFP. Ranking was performed in order of decrease in number of unique genes in each category. Calculation of unique gene quantity was performed manually with no taken into account degree of a category significance.

We used GO term annotations provided in the UniProtKB and InterPro databases to classify all retrieved proteins according to molecular functions and biological process categories (Figure 4). A total amount of terms can differ from the total amount of proteins aligned to each SLiM type of interest because (i) more than one GO term may be assigned to a unique protein and (ii) the same unique protein can belong to a variety of taxonomic categories. As shown in Figure 4A, metal ion binding, catalytic activity, and transferase activity were the predominant molecular function terms for AFP_14–20_-like motif-containing proteins. Additionally, there were proteins that exert oxidoreductase/electron transfer, DNA/RNA-binding, transcription factor, antimicrobial defense and immune response activities. The largest portion of proteins aligned to a GIP-9 segment of human AFP belonged to oxidoreductases and metal ion/iron-sulfur cluster binding, heme binding, and DNA binding proteins (Figure 4B).

Categorization of the retrieved proteins according to biological process terms showed that majority of AFP_14–20_-like motif-containing proteins are involved in transcriptional regulation, oxidative stress response, RNA processing, and host–pathogen defense response (Figure 4C). GIP-9-like motif-containing proteins were involved in aerobic respiration/electron transfer, response to environmental stress, metabolic process, regulation of gene expression, translation, DNA repair, and protein quality control (Figure 4D). 

Additionally, prominent roles belonged to proteins involved in response to the pathogen and immune response. There were apparent relationships between molecular function and biological process terms. For example, DNA binding and metal ion binding activities can be assigned to transcriptional regulation, while electron transfer/oxidoreductase activities and, partly, metal ion binding activity underlie cell response to oxidative stress and antimicrobial, antifungal, and antiviral defense responses. 

### 3.6. Prokaryotic Genes Are Required for Stress Tolerance

**Figure 5:**
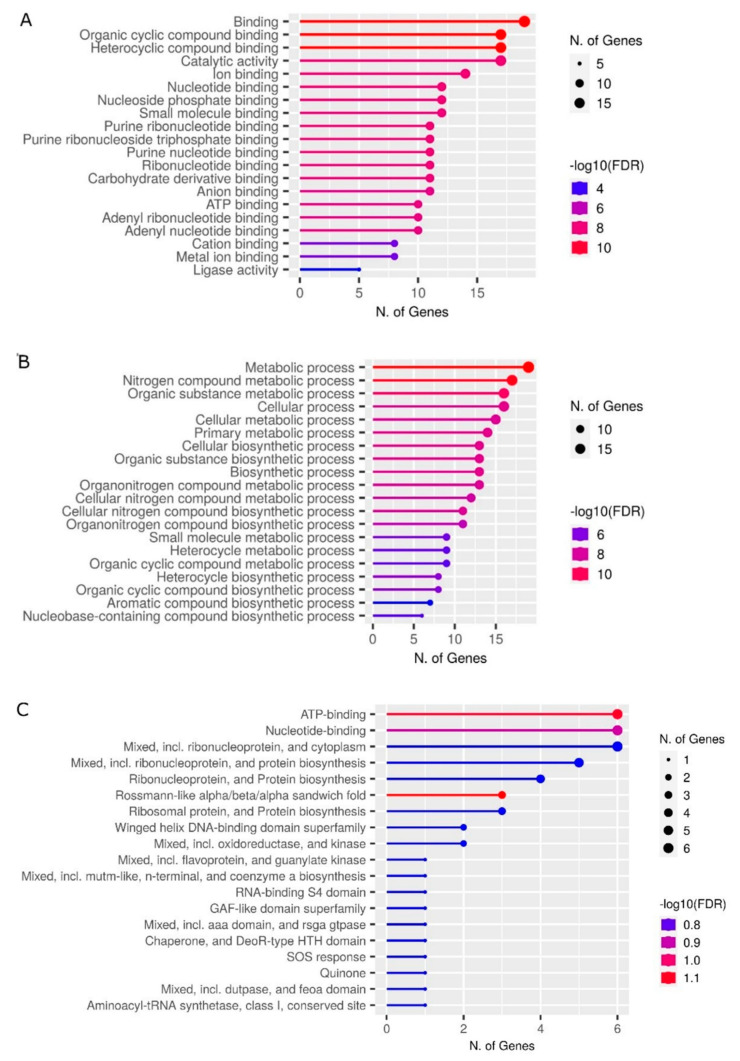
(**A**) Molecular function, (**B**) biological process, and (**C**) all-available gene set categorization in Gene Ontology terms of representative bacterial genome (*Acenitobacter* sp.). Lollipop chart at aspect ratio 1.5 and -log10 (FDR) heat maps for each category are shown. FDR is calculated based on nominal *p*-value from the hypergeometric test. FDR shows how likely the enrichment is by chance. Larger gene sets tend to have smaller FDR. N. of Genes indicates the number of genes for each category.

In order to identify the most statistically significant GO categories, we carried out gene set enrichment analysis with the use of ShinyGO v0.75 and gProfiler suites. In GO classification system, 389 unique genes encoding an AFP_14–20_-like motif containing proteins and 273 unique genes encoding a GIP-9-like motif containing proteins were mapped to the Ensembl gene IDs. Figure 5 depicts typical GO term-based functional categorization of genes encoding AFP_14–20_-like motif-containing proteins. From our gene set list, up to 41 bacterial genes were mapped to Ensembl genome IDs. 

As shown in Figure 5A, at FDR cutoff 0.2, bacterial genes associated with nucleotide/nucleic acid binding, ion/metal ion binding and ATP binding activities were retrieved at high statistical significance (low *p*-value) in molecular function categories. Not surprisingly, biological processes involved in metabolism and nucleotide/nucleic acid and amino acid biosynthesis required for bacterial reproduction were overrepresented (Figure 5B). However, when all available gene sets were retrieved, oxidoreductase and chaperone activity as well as chemical stimuli/stress response and SOS response activities were identified among statistically significant categories identified for bacterial proteins (Figure 5C). 

**Figure 6:**
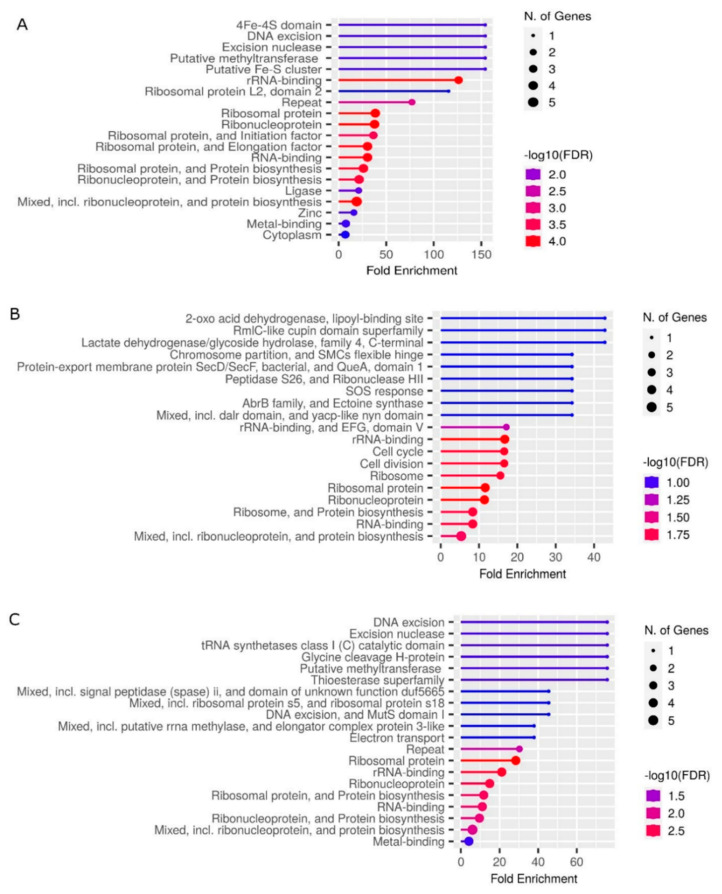
Prokaryotic genes coding for proteins containing GIP-9-like motifs. All-available gene set analysis of (**A**) *Desulfotomaculum guttoideum*, (**B**) *Bacillus selenitireducens*, and (**C**) *Clostridium aminophilum* genes. Categories are ranked by fold enrichment order; that is, the percentage of genes in the list belonging to each category divided by the corresponding percentage in the background. Fold enrichment indicates how drastically genes of a certain pathway are overrepresented. N. of Genes indicates the number of genes for each category.

These data were confirmed by functional enrichment analysis of genes encoding GIP-9-like motif-containing proteins. From our gene list, up to 29 unique genes were mapped to Ensembl genome IDs in each bacterial taxonomy. Figure 6A–C depicts the all-available gene set enrichment analyses for three representative bacterial species. As shown in Figure 6, pathways associated with metabolic processes, nucleic acid and protein biosynthesis, translation, and DNA repair are the most statistically significant. However, pathways that underlie cellular response to biotic and abiotic stress and chemical stimuli were identified. They included SOS response and oxidative stress response that occurs with the involvement of oxidoreductase/electron transfer enzymes including those containing Fe-S clusters.

### 3.7. Eukaryotic Genes Are Responsible for Stress and Defense Response

**Figure 7:**
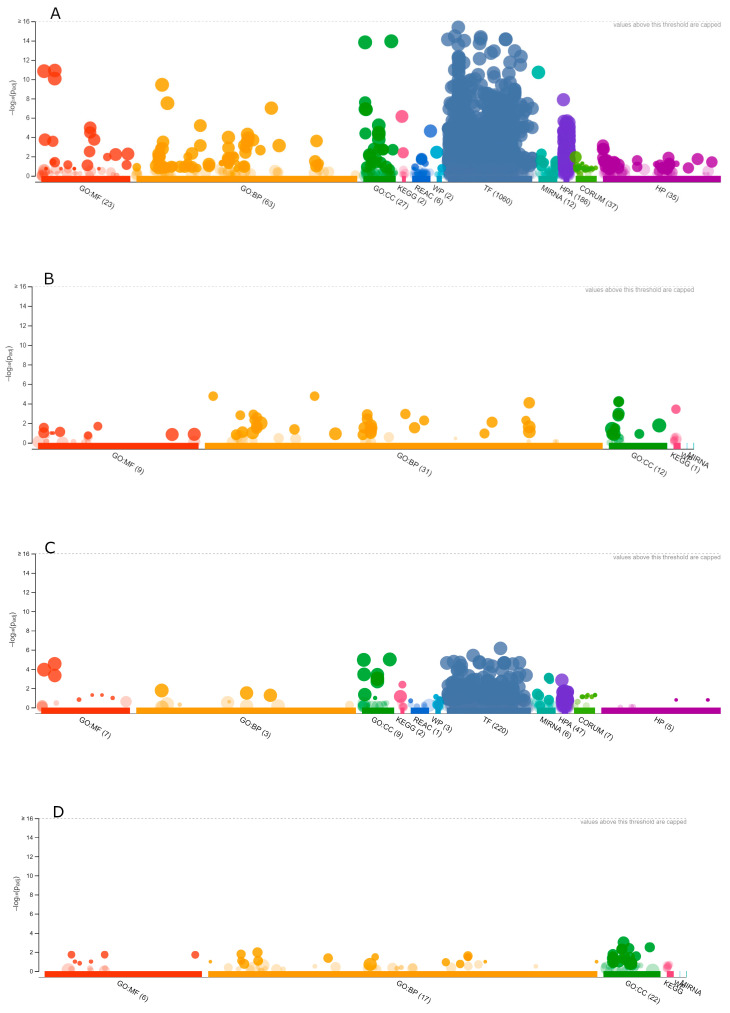
Grouping of eukaryotic genes. Manhattan plots of all *H. sapiens* (**A**,**C**) and *A. thaliana* (**B**,**D**) gene sets coding for (**A**,**B**) AFP_14–20_-like motif-containing and (**C**,**D**) GIP-9-like motif-containing proteins. The *x*-axis represents functional terms that are grouped and color-coded by data sources, while the *y*-axis shows the adjusted enrichment *p*-values in negative log10 scale. MF, molecular function; BP, biological process; CC, cellular component; KEGG, KEGG pathway; REAC, Reactome; WP, Wiki pathway; TF, transcription factor; MIRNA, microRNA; HPA, human Protein Atlas; CORUM, CORUM dataset; and HP, human phenotype. Each circle indicates the functional enrichment term, while the circle sizes correspond to the term size; larger terms have larger circles.

Figure 7 depicts the Manhattan plots that illustrate GO terms for human (A and C) and *A. thaliana* (B and D) gene sets coding for (A and B) AFP_14–20_-like motif-containing and (C and D) GIP-9-like motif-containing proteins. 

In humans, up to 54 unique genes encoding AFP_14–20_-like motif-containing proteins were mapped to Ensembl gene IDs. In other mammalians, the amount of corresponding unique genes constituted from 34 to 53 and from 14 to 20, respectively. 

**Figure 8:**
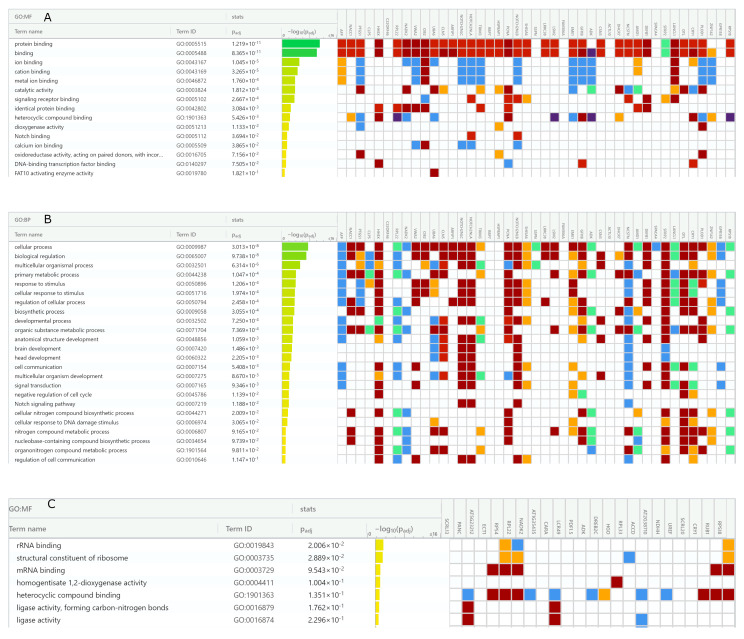

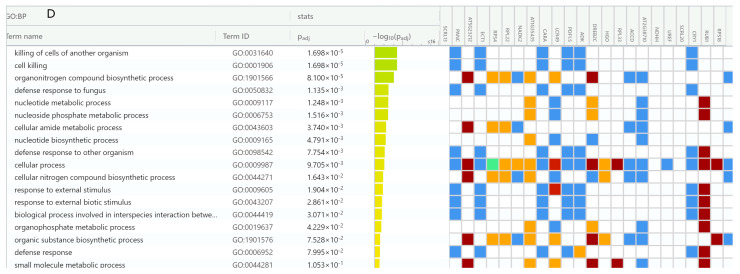
*H. sapiens* and *A. thaliana* gene set enrichment analysis of AFP_14–20_-like motif-containing proteins. Human genes categorized in (**A**) molecular function terms and (**B**) biological process terms. *A. thaliana* genes categorized in (**C**) molecular function terms and (**D**) biological process terms. Color codes indicate data inferred from: dark brown—experiment/direct assay, light brown—genetic and physical interactions, yellow—sequence similarity, dark purple—high throughput experiment, green—curator, blue—reviewed computational data.

In GO-based molecular function terms, *H. sapiens* protein/receptor binding, ion/metal ion-binding and calcium-binding, DNA and heterocyclic compound (nucleotide)-binding as well as dioxygenase and oxidoreductase activities were among the most significant categories (Figure 8A). As expected, in GO biological process terms, biosynthetic and developmental processes as well as cell communication and cell signaling pathways were identified as the most significant functional terms (Figure 8B). 

Unexpectedly, response to stress and chemical stimulus and DNA damage were also among statistically significant biological processes. This picture was typical for various animal species, where a wide range of stress response proteins including oxidoreductases, ubiquitin activating enzymes, channel activity regulators, and cell signaling proteins were retrieved. For example, in plants, up to 47 unique genes encoding AFP_14–20_-like motif-containing proteins were mapped to Ensembl gene IDs. These included proteins important for cell division such as those involved in RNA binding, nucleotide biosynthesis, and translation. Interestingly, those implicated in stress/defense response such as oxidoreductases and proteins involved in killing of other organisms were also among significant ones in plants (Figure 8C,D). 

**Figure 9:**
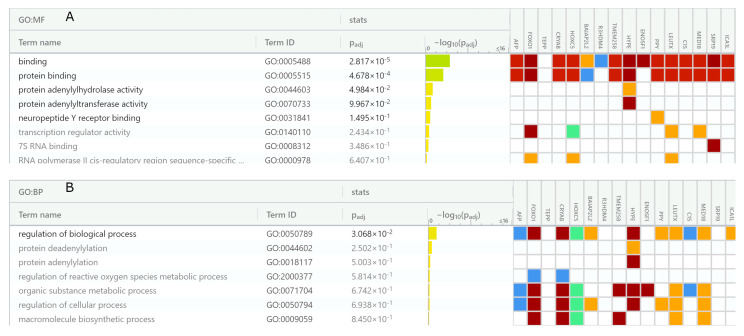

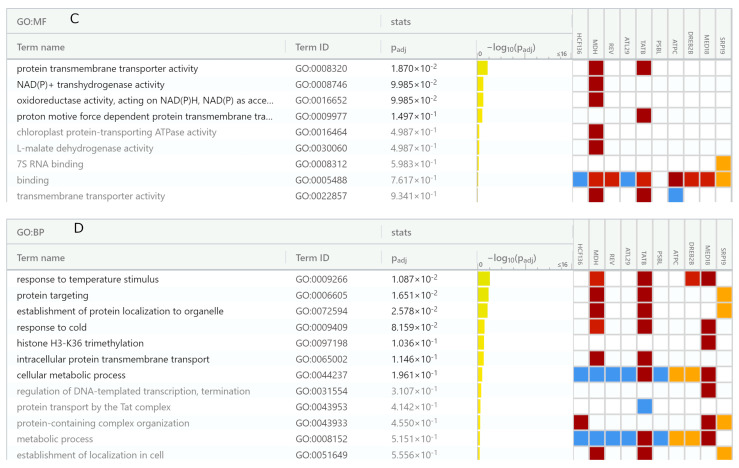
*H. sapiens* and *A. thaliana* gene set enrichment analysis of GIP-9-like motif-containing proteins. Human genes categorized in (**A**) molecular function terms and (**B**) biological process terms. *A. thaliana* genes categorized in (**C**) molecular function terms and (**D**) biological process terms. Color codes indicate data inferred from: dark brown—experiment/direct assay, light brown—genetic and physical interactions, yellow—sequence similarity, dark purple—high throughput experiment, green—curator, blue—reviewed computational data.

As for GIP-9-like motif-containing proteins, lower quantities of statistically significant GO terms were identified (Figure 9). Up to 21 unique genes in mammalians and up to 15 unique genes in plants were mapped to Ensembl gene IDs. 

In *H. sapiens* GO molecular function terms, protein and nucleotide binding activities along with chaperone and ion/metal ion binding activities were among overrepresented molecular function terms (Figure 9A). In biological process terms, immune and defense response as well as autophagy and apoptosis (Figure 9B) were identified among significant human genes. In plants, NADPH-dependent oxidoreductase, ion channel, and RNA/DNA binding activities, which underlie response to external stimulus, protein localization, and cellular metabolism were identified (Figure 9C,D).

## 4. Discussion

SLiMs are often found in the rapidly evolving intrinsically disordered regions of proteins and the motif acquisition can proceed through the convergent evolution [92]. Frequent mutations, small size, and low complexity make it difficult to identify motifs and to study their functions. Here, we used bioinformatics and GO enrichment analyses to search for SLiMs with sequence similarity to two AFP-derived sequences, LDSYQCT (AFP_14–20_) and EMTPVNPGV (GIP-9). We identified a vast array of similar motifs across all taxonomic categories including bacteria, viruses, archaea, and various eukaryotic species. 

One of the most prominent molecular functions of human and rodent AFPs is metal ion binding capability [93], which is similar to activities of majority of the retrieved in our study proteins. This capability underlies the involvement of proteins in various cellular processes including metabolism, transcriptional regulation, and redox regulation. Most of prokaryotic proteins were, unsurprisingly, involved in nucleotide, nucleic acid, amino acid, and protein biosynthesis necessary for their reproduction. However, the overwhelming majority of both prokaryotic and eukaryotic proteins including enzymes, transcription factors, quality control, and ribosomal proteins were involved in the cellular adaptation to environmental changes and various stress conditions. Our data suggest that AFP can use the SLiMs of interest to provide cellular adaptation to stress conditions during embryonic development and cancer growth.

### 4.1. AFP_14–20_-like Motif-Containing Proteins

We found that most bacterial and archaeal proteins containing short segments aligned with the AFP_14–20_ at high statistical significance (E-value of ~10^−5^–10^−4^) are involved in maintaining cellular redox balance (Table 1). For example, iron–sulfur (Fe-S) cluster proteins such as rubredoxins, ferredoxins, anaredoxin, and desulfoferrodoxin exert antioxidant activity and play important roles in bacterial adaptation to environmental changes [44,46]. These proteins have a unique structural characteristic of four Cys residues that surround the Fe-S clusters involved in electron transfer from cognate reductases to cytochrome P-450s enabling maintenance of the pathogen viability [35]. Fe-S clusters are found in many enzymes central to metabolic processes such as nitrogen fixation, respiration, and DNA processing and repair. Additionally, enzymes with flavin oxidoreductase activity such as choline dehydrogenase (Cdh), which oxidizes choline to betaine aldehyde for its further oxidation to betaine, were retrieved. Betaine is a source of CH_3_-group for biosynthesis of nucleotides, amino acids, etc., and provides adaptation of phototrophic bacteria to osmotic stress [53]. Choline oxidation is associated with electron transfer to the electron transportation chain (ETC) and ROS generation [38]. Reasonably, NADH-quinone oxidoreductase, ETC complex I, that is of the major sites of ROS production in many bacterial strains [65], was also aligned to AFP_14–20_ segment. Additionally, variety of modulators of environmental stress response were aligned to AFP_14–20_ segment. Histidine kinase response regulator protein, stress response protein YhaX [43,59], Sel1 domain-containing protein [42], and RagB/SusD family nutrient uptake outer membrane protein [39], which regulate host cell response to pathogen were among them. Moreover, bacterial 8-oxo-dGTP diphosphatase MutT and dITP/XTP pyrophosphatase enzymes, which are involved in SOS response due the removal of oxidatively damaged and non-canonical nucleotides, were retrieved [41].

Transcription factors that regulate gene expression in bacteria, archaea, and viruses for their adaptation to environmental stress conditions were also among the retrieved proteins. They included a helix-turn-helix domain-containing AraC family and a TetR transcriptional regulator that typically bind to target DNA and regulate pathogenic properties by sensing small molecule inducers such as urea, bicarbonate, and glycerol, etc. [49,52]. Bacterial ribosomal enzymes that catalyze posttranslational modification of proteins involved in translation were also aligned to the AFP_14–20_ segment. An example is *rimI* that encodes the ribosomal protein S18-alanine acetyltransferase [36]. Proteins involved in host–pathogen interaction via promoting nucleic acid replication and host adaptive immune response were found among viral proteins. They included host range factor 1 [48] and infected cell protein 47 (ICP47) [60], which function under redox changing. For example, ICP47 directly binds antigen-dependent transporter (TAP), leading to the occurrence of empty MHC-I that is under redox control due to disulfide bond oxidation/reduction [94]. 

In plants, the Rho family of Ras-related GTP-binding (Rop) proteins work as signaling switches that control growth, development and apoptosis in responses to various environmental stimuli [54]. A highly conserved catalytic PRONE (plant-specific Rop nucleotide exchanger) domain-containing proteins with strong substrate specificity for members of the Rop family were aligned to AFP_14–20_ segment. Additionally, developmental proteins with antimicrobial activity such as gibberellic acid-stimulated Arabidopsis (GASA) [66] were retrieved. There was also, though at low significance, the acidic leucine-rich nuclear phosphoprotein 32-related protein 2 involved in histone chaperone activity and the integration of environmental stress response in plants and immunomodulation and tumor progression in humans [95].

In animals, a variety of small proteins with ion channel regulator and toxin activity such as U-scoloptoxin [55], auger peptide hheTx2 [54], leiurutoxin-3 [62], and others produced by various mollusks, snakes, and insects were aligned with the AFP_14–20_ segment. Additionally, Cys-rich and metal ion binding small proteins including defensins, ranatuerins, and brevinines figure prominently in the alignment. These host defense proteins have key roles in oxidative stress response, immune response, and antimicrobial, antifungal, and antiviral activities [61]. Defensins have been implicated tumor growth exhibiting both tumor-suppressive and tumor-promoting effects [56]. In human carcinomas, defensins exert antitumor effects due to induction of apoptosis, inhibiting angiogenesis, and immunomodulation. 

Furthermore, transcription regulators that are involved in response to changes in microenvironmental conditions have been retrieved. They include CCHC-type domain-containing protein, C2H2-type zinc finger protein 142, nucleus accumbens-associated protein 1 (NAC1), and retinoic acid receptor RXR-gamma-B involved in various diseases including cancer and neurodevelopmental disorders [37,40,60,63]. Additionally, the importance of the extraction of calcium-binding EGF-like domain protein is that the AFP_14–20_ motif is a part of EGF and EGF-like domains involved in various signaling pathways [51]. Among them are JAG1/Notch signaling cascades, which activate a number of oncogenic factors that regulate cell proliferation, metastasis, angiogenesis, and drug-resistance [47]. Furthermore, denticleless protein homolog (DTL) has been associated with response to DNA damage and the immunosuppressive tumor microenvironment [45]. 

### 4.2. GIP-9-like Motif-Containing Proteins

As shown in Table 2, prokaryotic proteins containing sequences aligned with GIP-9 segment at high significance are preliminarily involved in maintaining genomic stability, transcriptional regulation, translation, and cell division. These included bacterial 2’-deoxycytidine-5’-triphosphate deaminase [67], forkhead-associated (FHA) domain-containing protein [68], chromosome partitioning protein ParA [69], AcrR family transcriptional regulator [70], dual specificity phosphatase [76], and glutamyl-Q tRNA(Asp) synthetase [79], as well as 30S and 50S ribosomal proteins [83]. They are involved in protection from DNA damage, genotoxicity, injury osmotic stress, etc. 

Additionally, thermonuclease family ribonuclease HII and PINc domain-containing proteins that are involved in DNA and RNA degradation under stress conditions to provide bacterial defense mechanism [75,89] were among the retrieved proteins. Furthermore, components of bacterial toxin–antitoxin systems such as addiction module HigA family antidote, which promote adaptation and persistence by modulating bacterial growth in response to stress [86], were also retrieved. 

Qualitatively, most bacterial proteins, including the cupredoxin domain-containing protein play pivotal roles in many metabolic pathways and regulation of redox homeostasis that are crucial for the pathogen survival [82]. NADPH-dependent oxidoreductases such as malate dehydrogenase and short-chain dehydrogenase (SDR) family oxidoreductase are among enzymes that undergo thiol group–redox switch for the involvement in adaptive response to oxidative stress conditions [78]. These also include a cytochrome c biogenesis protein that provides heme binding to apoprotein of cytochrome c and cytochrome c-552, the components of electron transfer and mitochondrial redox regulation [50,73]. Other proteins containing GIP-9-like segments involved in the pathogen response to oxidative stress included protein kinases and proteases. 

Many proteins, including those responsible for cell cycle control and embryonic development are regulated under oxidative stress conditions. For example, the Cys residue of CoA-binding protein can undergo S-thiolation in response to oxidative and metabolic stress [71]. Some of bacterial stress response protein homologs are implicated in disease pathogenesis in humans. For example, divalent cation tolerance protein CutA homolog has been proposed to mediate acetylcholinesterase activity and copper homeostasis, which are implicated in Alzheimer’s disease [87]. In proteobacteria, cell division proteins display redox transformation due to electron transfer and reduction of oxygen, nitrogen, and hydrogen sulfide [72]. Among viral proteins, the envelope glycoprotein E that is involved in host immune response to pathogen and viral protein kinase that have a role in virus virulence and tumor pathogenesis [77] were retrieved. 

Similar to AFP_14–20_-like motifs, GIP-9-like motifs were identified in small Fe-S cluster-containing proteins such as ferredoxins and Cisd2-a protein [80]. Furthermore, coevolution of bacteria with their hosts enabled them to tolerate oxidative stress conditions with the use of an antioxidant system (AOS) that includes both enzymatic and non-enzymatic components [96]. In this context, cupin domain-containing proteins contribute to counteracting the host defense due to functional diversity that includes an AOS component, the superoxide dismutase (SOD) enzyme [74]. Additionally, glutathione S-transferases play important roles in the environmental stress response due to S-glutathionylation of Cys residue and thiol groups resulting in target molecule detoxification [91]. 

In eukaryotes, GIP-9-like motifs were found in proteins such as small proline-rich proteins regulating cell cycle and cell proliferation and differentiation [85]. Interestingly, these proteins can be involved in tumor progression and their functioning is under redox control [97]. Additionally, ceruloplasmin, a major copper-carrying plasma protein that possesses ferroxidase activity and is involved in redox regulation [84], was retrieved. In plants and algae, photosystem II stability and assembly factor HCF136 that is essential for the formation of photosystem II complex and plastocyanin-like domain-containing protein that is involved in electron transfer during photosynthesis [81] were retrieved. They are regulated by redox switches between active–inactive states during light–dark transition [98]. Additionally, various stress-related proteins involved in quality control machinery including a C2H2-type zinc finger-containing protein and zinc metalloproteinases [85] were identified among GIP-9-like motif-containing proteins. Moreover, a variety of transmembrane proteins involved in ER stress response such as TMEM258 [99] and antimicrobial peptides, though at lower statistical significance, peptides were retrieved. 

In mammals, members of homeobox family transcription factors such as forkhead box protein O1 (FOXO1) and homeobox protein Hox-C5 (HOXC5) that play important roles in metabolism, cell proliferation, apoptosis, development, and stress resistance [90] were identified. Additionally, HSP family members, along with tumor necrosis factor (TNF) ligand family cytokines and Wnt-1 protein involved in Wnt/β-catenin signaling pathway, key players in redox regulation and cancer development [100], were among the retrieved proteins though at lower significance.

## 5. Conclusions

In our study, we undertook a comprehensive functional enrichment analysis of a wide range of proteins from all taxonomic groups and different functional classes. All these proteins have similar structural characteristics regarding the presence of conserved SLiMs. The both types of short sequences used as queries for sequence similarity search were derived from AFP, a major mammalian embryo-specific and tumor-associated protein. Therefore, the identification of a variety of transcription factors and proteins involved in cell signaling, cell cycle progression, cell proliferation and differentiation, and protein quality control was anticipated. However, unexpectedly, various prokaryotic and eukaryotic proteins responsible for cellular response to both biotic and abiotic stress were retrieved as containing the both AFP_14–20_-like and GIP-9-like motifs. They included proteins implicated in the adaptation and protection against pathogens, reactive oxygen species, toxins, and various chemical agents. Moreover, the overwhelming majority of retrieved transcription factors and proteins involved in replication and translation were reported to participate in cellular and organismal adaptation environmental stress stimuli. 

We hypothesized that both the AFP-derived peptides can arise from prototype peptides during the long evolutionary time. At the early stages of biochemical evolution, these peptides were involved in cellular stress response and preserved this function in modern proteins, including AFP. Therefore, bioinformatics and GO functional enrichment analyses of SLiMs allows insight into the common functions of a variety proteins and the involvement of AFP in cellular response to external and internal stimuli during embryonic development and cancer growth. Nevertheless, our data require further confirmation with the use of experimental approaches.

## Figures and Tables

**Table 1:** Representative proteins retrieved from the UniProtKB database as containing AFP_14–20_-like motifs (at E-value ˂ 0.05).

Protein Name	Species	Entry Code	Gene Symbol	Alignment	Aa Positions	Identity Degree	E-Value	Biological Roles	Reference
Rubredoxin	*Methanoregulaceae archaeon*	TR: A0A1V5A688	*rub_2*	LDSYQCT MDSYQCT	1–7	85.7%	3.9 × 10^−11^	Electron transfer, iron-binding, redox regulation	[35]
Ribosomal protein S18-alanine N-acetyltransferase	*Acinetobacter* sp.	TR: A0A5C8C7V6	*rimI*	LDSYQCT LDSYQCT	1–7	100%	1.5 × 10^−9^	Translational regulation	[36]
CCHC-type domain-containing protein	*Crassostrea gigas*	TR: K1QN18	*CGI*	LDSYQCT MDSYQCS	11–17	71.4%	3.4 × 10^−6^	Transcriptional regulation, response to environmental changes	[37]
Choline dehydrogenase	*Comamonadaceae bacterium*	TR: A0A2H0JD95	*COW02_01535*	LDSYQCT LDSYQCT	134–140	100%	5.3 × 10^−6^	Oxidative stress response	[38]
RagB/SusD family nutrient uptake outer membrane protein	*Ginsengibacter hankyongi*	TR: A0A5J5IHS1	*FW778_00440*	LDSYQCT LDSYQCT	303–309	100%	5.3 × 10^−6^	Host cell response to pathogen	[39]
Zinc-finger protein 142,	*Nothobranchius kuhntae*	TR: A0A1A8JQF5	*ZNF142*	LDSYQCT LDSYRCS	24–30	71.4%	8.8 × 10^−6^	DNA-binding, response to environmental changes	[40]
Ferredoxin-type protein NapF	*Salipiger* sp.	TR: A0A2A3JNE6	*CLG85_24025*	LDS YQCT LDSAQCT	3–9	85.7%	1.1 × 10^−5^	Nitrate oxidation, redox balance	[35]
8-oxo-dGTP diphosphatase MutT	*Spirochaetae bacterium*	TR: A0A2N1RAE0	*MutT* *(CVV52_19070)*	LDS YQCT MDAYQCT	81–87	71.4%	1.5 × 10^−5^	Removal of oxidatively damaged guanine, DNA repair	[41]
Sel1 domain protein repeat-containing protein	*Nitrosococcus halophilus*	TR: D5BUP3	*Nhal_0240*	LDSYQCT LDGYQCT	63–69	85.7%	3.0 × 10^−5^	Protein degradation, response to pathogen	[42]
Histidine kinase response regulator	*Bacteroidetes bacterium*	TR: A0A2M7KDX5	*COZ59_01780*	LDS YQCT MDGYQCT	45–51	71.4%	3.8 × 10^−5^	Regulation of stress response	[43]
Ferredoxin	*Candidatus electrothrix aarhusiensis*	TR: A0A444IS91	*H206_03280*	LDSYQCT I DTYQCS	6–12	57.1%	5.5 × 10^−5^	Electron transfer, metal ion binding, redox regulation	[44]
DTL protein	*Balaeniceps rex*	TR: A0A7L2U6N2	*Dtl*	LDSYQCT LDSYQCS	10–16	85.7%	5.8 × 10^−5^	Response to DNA damage and immunosuppressive microenvironment	[45]
Anaredoxin	*Nostoc* sp.	SP: Q44141	*Adx*	LDSYQCT LESYQCM	19–25	71.4%	6.1 × 10^−5^	Oxidoreductase, endonuclease, redox regulation	[46]
Protein jagged-1	*Trichoplax* sp. *H2*	TR: A0A369RNS5	*TrispH2_012046*	LDSYQCT LDQYQCT	207–213	85.7%	1.1 × 10^−4^	Notch signaling, angiogenesis, response to hypoxia	[47]
Host range factor 1	*Lymantria dispar multicapsid nuclear polyhedrosis virus*	SP: Q90165	*HRF-1*	LDSYQCT VDSYKCT	14–20	71.4%	1.6 × 10^−4^	Host response to virus	[48]
Helix-turn-helix domain-containing protein	*Cytophagaceae* *bacterium*	TR: A0A4Q3N6Z7	*EOO38_22880*	LDSYQCT LDDYQCT	59–65	85.7%	2.4 × 10^−4^	DNA binding, response to pathogen	[49]
Cytochrome c	*Ignavibacteriae bacterium*	TR: A0A660Z7I5	*DRQ13_06320*	LDSYQCT LDTYQCT	239–245	85.7%	2.9 × 10^−4^	ETC component, oxidative stress	[50]
Calcium binding EGF domain protein	*Trichinella nativa*	TR: A0A1Y3EHZ0	*D917_09763*	LDSYQCT MDSYQCR	79–85	71.4%	2.9 × 10^−4^	Cell proliferation and adhesion, angiogenesis under hypoxia	[51]
TetR family transcriptional regulator	*Pedobacter duraqua*	TR: A0A4V3C417	*CLV32_0466*	LDSYQCT LDSYQCK	73–78	85.7%	3.4 × 10^−4^	Sensing small molecule inducers	[52]
Flavin oxidoreductase	*Salinivibrio sharmensis*	TR: A0A1V3GXS2	*BZG19_13810*	LDSYQCT LDSYHCT	188–194	85.7%	4.0 × 10^−4^	Oxidative stress response	[53]
PRONE domain-containing protein	*Prunus persica* *Prunus armeniaca*	TR:M5VXJ9	*PRUPE_ppa002319mg*	LDSYQCT MDSYQCT	666–672	85.7%	4.5 × 10^−4^	Response to environmental stimuli	[54]
U-scoloptoxin(05)-Er1a	*Ethmostigmus rubripes*	SP: P0DPX8	*N/A*	LDSYQCT LECYQCT	21–27	71.4%	7.1 × 10^−4^	Toxin activity, defense response	[55]
Fungal defensin eurocin	*Aspergillus amstelodami*	SP: K7NSL0	*N/A*	LDSYQCT GDAYQCS	6–11	57.1%	9.2 × 10^−4^	Antimicrobial peptide, defense response	[56]
Yemanuclein	*Drosophila melanogaster*	SP: P25992	*yem*	LDSYQCT LDDYQCT	846–852	85.7%	1.0 × 10^−3^	DNA binding, chromatin assembly, genome stability	[57]
dITP/XTP pyrophosphatase	*Legionella* *pneumophila*	SP: Q5X245	*lpp2548*	LDSYQCT LNEYQCT	160–166	71.4%	5.5 × 10^−3^	Preventing non-canonical nucleotide incorporation, SOS response	[41]
Augerpeptide hheTx2	*Hastula hectica*	SP: P0CI09	*N/A*	LDSYQCT SDSCQCT	11–17	71.4%	8.7 × 10^−3^	Toxin activity, C-rich antimicrobial peptide	[58]
Stress response protein YhaX	*Bacillus subtilis*	SP: O07539	*yhaX*	LDSYQCT LESYQCN	96–102	71.4%	8.9 × 10^−3^	Mg and Cu ion binding, response to stress	[59]
Nucleus accumbens-associated protein 1	*Mus musculus*	SP: Q7TSZ8	*Nacc1*	LDSYQCT LDSVQCT	172–178	85.7%	9.2 × 10^−3^	Response to hypoxic microenvironment	[60]
Ranatuerin-3	*Lithobates catesbeianus*	SP: P82780	*N/A*	LDSYQCT LDKIKCT	18–24	57.1%	1.3 × 10^−2^	Host antimicrobial response	[61]
Leiurutoxin-3	*Leiurus* *quinquestriatus*	SP:P45661	*N/A*	LDSYQCT YDSSQCE	8–14	57.1%	1.5 × 10^−2^	K^+^-channel regulator, defense response	[62]
Retinoic acid receptor RXR-gamma-B	*Danio rerio*	SP:Q6DHP9	*rxrgb*	LDSYQCT MSSYQCT	112–118	71.4%	1.8 × 10^−2^	Gene expression and immune response	[63]
Infected cell protein 47	*Human herpesvirus 2*	SP: P14345	*US12*	LDSYQCT LDSSRCT	12–18	71.4%	2.4 × 10^−2^	Inhibiting CD8+ host adaptive immune response	[64]
NADH-quinone oxidoreductase subunit A	*Roseiflexus* sp. *Azotobacter vinelandii*	SP: A5UXK0	*nuoA*	LDSYQCT LDTYECG	39–45	57.1%	2.7 × 10^−2^	Electron transfer oxidative stress response	[65]
Brevinin-2Re	*Pelophylax ridibundus*	SP: C0HKZ9	*N/A*	LDSYQCT LDK IQCK	18–24	57.1%	3.0 × 10^−2^	Antimicrobial defense response	[61]
Gibberellin-regulated protein 6	*Arabidopsis thaliana*	SP: Q6NMQ7	*GASA6*	LDSYQCT LKSYQCG	38–44	71.4%	4.7 × 10^−2^	Plant development, antimicrobial response	[66]

**Table 2:** Representative proteins retrieved from the UniProtKB database as containing GIP-9-like motifs (at E-value ˂ 0.05).

Protein Name	Species	Entry Code	Gene Symbol	Alignment	Aa Positions	Identity Degree	E-Value	Biological Role	Reference
2′-deoxycytidine 5’-triphosphate deaminase	*Parvularcula* sp.	TR: A0A357L903	*DEA40_15450*	EMTPVNPGV EMTP I NPGL	184–192	77.8%	2.2 × 10^−7^	Maintaining dNTP pool and genomic stability	[67]
FHA domain-containing protein	*Cryobacterium* sp.	TR: A0A6H3K8T7	*E3O68_01825*	EMTPVNPGV ERTPVNPGV	64–72	88.9%	9.1 × 10^−7^	DNA damage response, innate immune response	[68]
Chromosome partitioning protein ParA	*Verrucomicrobiaceae bacterium*	TR: A0A4Q3BDS1	*EOP84_15500*	EMTPVNPGV EMTPFNPGL	70–78	77.8%	7.4 × 10^−6^	Chromosome partitioning and segregation	[69]
AcrR family transcriptional regulator	*Gordonia humi*	TR:A0A840EPS7	*BKA16_000043*	EMTPVNPGV EMSPVDPGV	158–166	77.8%	3.1 × 10^−5^	Transcriptional regulation, resistance to toxic chemicals	[70]
CoA-binding protein	*Acidocella* sp.	TR: A0A257Q4I9	*B7Z75_09205*	EMTPVNPGV EVTPVNPGL	42–50	77.8%	3.2 × 10^−5^	Cellular metabolism under redox control	[71]
Cell division protein FtsL	*Betaproteobacteria bacterium*	TR:A0A2N2UB63	*ftsL*	EMTPVNPGV KMRPVNPGI	72–80	66.7%	9.2 × 10^−5^	Chromosome scaffolding, cell cycle, Zn^2+^ ion sensitivity	[72]
Cytochrome c-type biogenesis protein CcmE	*Agrobacterium fabrum*	SP: Q8UGR1	*ccmE*	EMTPVNPGV EKTPVNPGT	43–51	77.8%	1.2 × 10^−4^	Apoprotein-heme interaction, redox response	[73]
Cupin domain-containing protein	*Bradyrhizobium* sp.	TR: A0A525IHT2	*E7774_03250*	EMTPVNPGV E I TPVGPGV	75–83	77.8%	5.3 × 10^−4^	Response to biotic and abiotic stress; SOD activity	[74]
Thermonuclease family protein	*Chloroflexia bacterium*	TR: A0A7W0PLY7	*H0T93_01160*	EMTPVNPGV E I TPVNPG I	126–134	77.8%	5.9 × 10^−4^	DNA and RNA degradation, defense response	[75]
Dual specificity phosphatase	*Dicentrarchus labrax*	TR: A0A8C4HFW9	*N/A*	EMTPVNPGV NLTPVNPGV	25–33	77.8%	6.9 × 10^−4^	Cell signaling, protection from genotoxicity, and injury	[76]
Envelope glycoprotein E	*Varicella-zoster virus*	SP: P09259	*gE*	EMTPVNPGV E ITPVNPGT	524–532	77.8%	7.0 × 10^−4^	Viral immune response	[77]
NADPH-dependent oxidoreductase	*Brevibacterium aurantiacum*	TR: A0A4Z0KM68	*EB834_09640*	EMTPVNPGV RMTPVSPGV	135–143	77.8%	3.7 × 10^−3^	Oxidative stress response	[78]
Ferredoxin	*Planctomycetes* *bacterium*	TR: A0A3L7UG95	*DWI22_14495*	EMTPVNPGV EMSPLCPG I	43–51	55.6%	4.8 × 10^−3^	Iron-sulfur cluster binding, redox response	[35]
Glutamyl-Q tRNA(Asp) synthetase	*Gammaproteobacteria bacterium*	TR: A0A4Y8UV00	*gluQ*	EMTPVNPGV ELRPVNPGV	17–25	77.8%	4.6 × 10^−3^	Response to amino acid availability	[79]
Cisd2-a protein	*Symbiodinium pilosum*	TR: A0A812YDJ7	*cisd2-a*	EMTPVNPGV KPTPVNPG I	31–39	66.7%	5.6 × 10^−3^	Electron transfer, redox response	[80]
Plastocyanin-like domain-containing protein	*Strigops habroptila*	TR: A0A672VBQ4	*N/A*	EMTPVNPGV EMSPENPGT	23–32	66.7%	7.2 × 10^−3^	Electron transfer regulated by light-dark switches	[81]
Cupredoxin domain-containing protein	*Thermoleophilaceae bacterium*	TR: A0A838PN94	*H0U20_06955*	EMTPVNPGV ELNPANPGV	37–45	66.7%	9.7 × 10^−3^	Oxidative stress response	[82]
50S ribosomal protein L13e	*Aeropyrum pernix*	SP: Q9YEN9	*rpl13e*	EMTPVNPGV KLGPVDPGV	15–23	55.6%	1.1 × 10^−2^	Ribosome assembly	[83]
Ceruloplasmin	*Pterocles gutturalis*	TR: A0A093CDT1	*CP*	EMTPVNPGV EMTPQNPGT	166–174	77.8%	1.1 × 10^−2^	Copper-binding ferroxidase activity	[84]
Proline-rich protein 2	*Lottia gigantea*	SP: B3A0R8	*PRH2*	EMTPVNPGV PMSPVRPGV	90–98	66.7%	1.1 × 10^−2^	Cell cycle regulation under redox control	[85]
Addiction module HigA family antidote	*Thiogranum longum*	TR: A0A4R1H8U5	*DFR30_1540*	EMTPVNPGV KLTP IHPGV	4–12	55.6%	1.7 × 10^−2^	Plasmid addiction, bacterial growth under stress conditions	[86]
Divalent-cation tolerance protein CutA	*Actinomadura rudentiformis*	TR: A0A6H9YAH5	*F8566_39675*	EMTPVNPGV EVTPGNPGV	10–18	77.8%	1.3 × 10^−2^	Response to Cu^2+^ ion	[87]
Ribonuclease HII	*Rhodanobacter* sp.	TR: A0A522L7A0	*rnhB*	EMTPVNPGV ELTPANPGL	3–11	66.7%	1.7 × 10^−2^	RNA binding, defense response	[88]
C2H2-type domain-containing protein	*Gibberella nygamai*	TR: A0A2K0W957	*FNYG_07693*	EMTPVNPGV EPTPVNPGL	133–141	77.8%	2.0 × 10^−2^	Transcriptional regulation, response to environmental changes	[89]
Forkhead box protein O1	*Bos taurus*	SP: E1BPQ1	*FOXO1*	EMTPVNPGV IMTPVDPGV	445–453	77.8%	2.8 × 10^−2^	Metabolic homeostasis under oxidative stress	[90]
Glutathione S-transferase	*Caulobacter vibrioides*	TR: A0A258CQ14	*B7Z12_21310*	EMTPVNPGV EMI PVN IGV	30–38	77.8%	3.8 × 10^−2^	Substrate S-glutathionylation and detoxification	[91]

## Data Availability

Data are available in a publicly accessible repository. The data presented in this study are openly available in FigShare at https://doi.org/10.6084/m9.figshare.20456727 accessed on 21 December 2022.
